# Title I: Modernizing Definitions and Programs Under the Administration on Aging

**DOI:** 10.1093/geroni/igaa057.2518

**Published:** 2020-12-16

**Authors:** Marie Gualtieri

**Affiliations:** Washington, District of Columbia, United States

## Abstract

The recent reauthorization of the Older Americans Act adds language and definitions to current issues facing the aging population. Specifically, Title I includes definitions related to program adaptation and coordination, workforce and long-term care issues, nutrition and social isolation, as well as family caregivers. Different from the last authorization, these definitions span beyond the individual experience to include other entities impacted by an aging society, such as the workforce and families. Overall, the Title I reauthorization seeks to modernize policy to reflect the current influx of the older adult population and its consequences.

